# BRP-Net: A discrete-aware network based on attention mechanisms and LSTM for birth rate prediction in prefecture-level cities

**DOI:** 10.1371/journal.pone.0307721

**Published:** 2024-09-12

**Authors:** Mingfu Xue, Junyu Zhu, Rusheng Wu, Xiayiwei Zhang, Yuan Chen

**Affiliations:** 1 Johns Hopkins University, Baltimore, Maryland, United States of America; 2 College of Electrical and Information Engineering, Hunan University, Changsha, China; 3 College of Finance and Statistics, Hunan University, Changsha, China; Abu Dhabi University, UNITED ARAB EMIRATES

## Abstract

The continuous decline in the birth rate can lead to a series of social and economic problems. Accurately predicting the birth rate of a region will help national and local governments to formulate more scientifically sound development policies. This paper proposes a discrete-aware model BRP-Net based on attention mechanism and LSTM, for effectively predicting the birth rate of prefecture-level cities. BRP-Net is trained using multiple variables related to comprehensive development of prefecture-level cities, covering factors such as economy, education and population structure that can influence the birth rate. Additionally, the comprehensive data of China’s prefecture-level cities exhibits strong spatiotemporal specificity. Our model leverages the advantages of attention mechanism to identify the feature correlation and temporal relationships of these multi-variable time series input data. Extensive experimental results demonstrate that the proposed BRP-Net has higher accuracy and better generalization performance compared to other mainstream methods, while being able to adapt to the spatiotemporal specificity of variables between prefecture-level cities. Using BRP-Net to achieve precise and robust prediction estimates of the birth rate in prefecture-level cities can provide more effective decision-making references for local governments to formulate more accurate and reasonable fertility encouragement policies.

## Introduction

In recent years, the birth rate in China has been declining year by year. According to the 2020 Seventh National Census Report, the new birth population in China was 12 million, with a birth rate of only 8.5‰, reaching a historic low. Furthermore, predictions indicate a sharp decrease in China’s population size starting from 2050. The disappearance of demographic dividend will lead to China losing its comparative advantage in the global economic market and weakening its comprehensive national strength [1]. Moreover, the continued decline in birth rate will further accelerate population aging in China, accompanied by a series of social issues that will hinder development, such as population crisis [2], labor shortages [3], declining productivity, increased household burdens [4], decreased GDP growth rates [5], and reduced innovation effects [6]. As overall national policy documents are unable to swiftly address the issue of declining birth rates, formulating differentiated policies to stimulate birth rates based on their unique characteristics may be one way to address the continued decline in birth rates. Therefore, accurate prediction and analysis of birth rates, especially at the prefecture level, hold significant theoretical and practical value.

Currently, most methods used in the industry to predict regional birth rates are based on the Auto-regressive Integrated Moving Average (ARIMA) model. The ARIMA model primarily uses stationary output variables and multiple input variables to construct the model, and it is a statistical model used for time series analysis and forecasting. It combines Autoregressive (AR) model, Integrated (I) model, and Moving Average (MA) model to capture trends and seasonal variations in time series data. In predicting birth rates, the ARIMA model can be used to build a model using historical birth rate data to capture seasonal changes and long-term trends in birth rates, thus enabling the prediction of future birth rates. H et al. [7] accurately predicted the birth rates in the United States from 2002 to 2008 using the ARIMA model based on birth rate data from 1975 to 2001. Guo Jing et al. [8] used the ARIMA method to predict the total population of Yunnan Province for 8 periods from 1973 to 2018. Hasan et al. [9] constructed a multiple stepwise linear regression model to predict the birth rate in Bangladesh. Buah et al. [10] used a Box-Jenkins method based on the ARIMA model to model and forecast monthly birth rates in Ghana. However, these methods rely too much on manual prior knowledge and are unable to extract deep clues and coupling relationships between variables, resulting in overall low prediction accuracy and limited time span.

With the rapid advancement of machine learning, research on population birth rate prediction based on machine learning and neural network technologies is gradually becoming mainstream. Population birth rate prediction based on traditional machine learning typically involves feature engineering, which entails selecting and constructing features related to birth rates, such as age structure, educational level, and economic development. Subsequently, regression analysis, decision trees, or support vector machines are used for modeling and prediction. These methods are advantageous due to their ease of interpretation and implementation, and are suitable for relatively simple data patterns. Otoom et al. [11] compared the performance of 17 machine learning techniques in predicting population growth rates in the United Nations dataset, with random forest outperforming all other techniques in terms of predictive performance and robustness to missing features. However, traditional machine learning methods are unable to accurately extract coupled features among multiple variables and tend to have excessive parameters, thereby lacking precision in predicting complex population trends.

Furthermore, methods for predicting population birth rates based on deep learning primarily involve the use of recurrent neural networks (RNN), long short-term memory networks (LSTM), and convolutional neural networks (CNN). These methods are characterized by their ability to handle time series data and complex nonlinear relationships, while also possessing automatic feature extraction capabilities. Jia et al. [12] and Wu et al. [13] used BP neural networks to predict birth rates by constructing multiple fully connected layers to automatically analyze and learn coupled relationships among multiple time series variables. Viktoria et al. [14] utilized an ANN long short-term memory model (LSTM) to forecast population and birth rates for various counties in Alabama, USA. However, traditional CNN and LSTM-based methods can to some extent handle time series data and perform automatic feature extraction. Nevertheless, when confronted with complex research subjects such as China’s prefecture-level cities, which exhibit regional specificity and involve highly coupled development variables, these methods still struggle to accurately extract the temporal features of the data, thereby indirectly affecting the predictive accuracy of future population birth rates.

Based on the aforementioned research background and methodological issues, this paper proposes a BRP-Net network for predicting the population birth rate of China’s prefecture-level cities. Considering that population birth rate prediction remains a challenge based on multidimensional time series variables, and the input variable data from various prefecture-level cities exhibit regional specificity and local sparsity, inspired by [15], we employ Long Short-Term Memory (LSTM) networks as the backbone. Additionally, the input of the proposed BRP-Net network consists of mixed features of 12 factors most relevant to the population birth rate of prefecture-level cities, covering aspects such as economy, education, healthcare, and population structure. We design a dual-branch attention module containing feature attention and temporal attention to obtain high-quality temporal feature representations [16, 17], enhancing the traditional LSTM, thus achieving improved predictive accuracy and effectively handling the regional-specific data features of population birth rates in prefecture-level cities for accurate and robust prediction. The main contributions of this paper are as follows:

1)We propose a discrete-aware deep learning network framework BRP-Net based on the two-branch attention mechanism and LSTM for predicting the birth rate of Chinese prefecture-level cities. The network can efficiently extract long-range historical time-series features of population composite data from discrete prefecture-level cities in China to achieve robust and accurate birth rate prediction.

2)We explore and analyze the spatial linear correlation of the 12 most relevant multidimensional factors to the population birth rates in prefecture-level cities, as well as their significance in predicting the birth rate outcome. Visual experimental results are provided to assist prefecture-level governments in implementing precise policies.

3)We introduce a novel indicator, PBQ, for measuring the impact of policies on population birth rates, along with a regional calculation method for this indicator. Extensive comprehensive experimental results reveal that, compared to traditional models, the proposed PBQ indicator helps address the specificity of multivariate data at the regional level and significantly improves the accuracy of prediction results.

The remainder of this paper is organized as follows: Section 2, Related Work, primarily introduces the factors affecting population birth rates, mainstream population birth rate prediction methods, and research on the application of LSTM and attention mechanisms in handling multidimensional time series issues. Section 3 provides a detailed explanation of the proposed BRP-Net method and introduces the calculation method for the PBQ indicator. Section 4 presents experimental results comparing the proposed method with other mainstream methods on a dataset we collected and organized. Section 5 summarizes and discusses the paper.

## Related work

### Social studies on birth rates

Regarding the economic and social effects of low fertility rates, we found that the sustained decline in birth rates has brought adverse effects to economic and social development, such as population crisis, labor shortage, declining productivity, and increased social burden of care. Firstly, low fertility rates have self-reinforcing mechanisms in demography, sociology, and economics that may lead to a “low fertility trap” with population crises [2]. Secondly, the decrease in fertility rates implies a reduction in the labor force [18], leading to the Lewis turning point and the disappearance of demographic dividends [3]. Additionally, the aging of population, especially the aging of the workforce further reduces labor productivity, hindering industrial structural upgrading and technological progress. [6] Maesta et al.’s study [5] also shows that two-thirds of the total impact of aging on per capita GDP growth comes from the slowdown in labor productivity growth and one-third from the slowdown in labor force growth. Aging is accompanied by innovation effects and labor force effects. As the degree of aging deepens, the innovation effect decreases while the labor force effect strengthens, which is unfavorable for economic development [19]. Lastly, the decrease in fertility rates means an increase in the elderly dependency ratio, further exacerbating the social financial burden [4]. Zheng and Rui’s study [20] using theoretical models and provincial panel data in China also found that an increase in the elderly dependency ratio is unfavorable for economic growth.

Regarding factors affecting birth rates, we focus on the impact of economic development on fertility, combining education, housing prices, and female labor participation factors. With the impact of economic development on fertility intentions, scholars generally believe that there is a negative correlation between fertility rates and economic development levels [21, 22], as the utility of having children decreases as family income decreases, while the cost of raising children continues to rise. Therefore, families in highly developed economies have fewer children and invest more in each child’s education [23]. Murat, F et al. [24] linked fertility, economic development and human capital and found that economic development increases the stock of human capital and raises the opportunity cost of childbearing, thus inducing individuals to delay childbearing.

Education mainly affects fertility rates in three ways: first, an increase in educational attainment means longer years of study, which delays women’s childbearing age and reduces fertility rates [25, 26]. Second, an increase in average years of education in society raises children’s education costs and lowers fertility rates [27]. Finally, an increase in female education raises the opportunity cost of having children, delaying childbirth and reducing fertility rates [28].

Housing prices also have a significant impact on fertility intentions. On the one hand, housing costs compete with childcare costs and squeeze out fertility [29]. Couples who practice family planning may delay childbirth due to a lack of suitable housing [30]. On the other hand, rising housing prices bring wealth effects that increase fertility rates. Dettling et al.’s study [31] on the income and crowding-out effects of rising housing prices found different impacts on homeowners and non-homeowners.

Female labor participation affects fertility rates from two aspects: on the one hand, an increase in female labor participation and wages raises household income and increases fertility rates; on the other hand, an increase in female labor participation means that the “shadow price” of having children rises, lowering fertility rates. Most studies show that as women’s income increases, childbirth occurs later and fertility levels decrease [32]. Some studies show that with improved childbirth support measures and women’s status, an increase in female wages can bring “income effects” and raise fertility rates [33].

In addition to the aforementioned reasons, population mobility has a negative impact on fertility desire by increasing the cost of living and influencing marriage and childbearing habits, particularly in the process of rural population migration to urban areas [34]. Furthermore, the social support system, mainly comprised of medical and social security levels, also affects the birth rate. Wang et al. [35] used the CHNS database to study the influence of new rural cooperative medical insurance on fertility desire. The study showed that social security has a crowding-out effect and an income effect on fertility, and empirical evidence indicates that the crowding-out effect is dominant, which decreases residents’ fertility desire. Holmqvist et al. [36] also found a negative impact of pension coverage on the fertility rate using partial national data from South Africa.

In summary, this paper used comprehensive data covering the aforementioned indicators, encompassing 12 dimensions of urban development in prefecture-level cities, as input variables for BRP-Net. However, in addition to the above indicators, policies are also an important factor influencing population birth rates in China. Therefore, we have proposed a policy-induced birth impact indicator, PBQ, and provided calculation methods for different regions. Subsequent experimental results have also demonstrated the effectiveness of this indicator.

### The studies on birth rate prediction

In the past few decades, scholars have proposed various methods for forecasting birth rates, including traditional time series analysis, machine learning, and deep learning methods. Traditional time series analysis methods include ARIMA and VAR models, which are capable of handling non-stationary time series data and making relatively accurate predictions of future trends over a certain period. For instance, H et al. [7] accurately predicted the birth rates in the United States from 2002 to 2008 based on data from 1975 to 2001, using an ARIMA-based method. Guo et al. [8] used the ARIMA method to forecast the total population of Yunnan Province from 1973 to 2018. Hasan et al. [9] constructed a multivariate stepwise linear regression model to forecast the birth rates in Bangladesh. Wang et al. [37] used a vector autoregressive model to forecast birth rates in China and discussed its feasibility in the Chinese healthcare sector. Collantes-duarte et al. [38] compared three time series forecasting techniques, including traditional ARIMA statistical forecasting methods, artificial neural networks, and emerging intelligent methods such as Neo Fuzzy Neurons, to forecast birth rates in Spain. However, birth rates are influenced by various factors such as social policies, economic development, and cultural changes, which may extend beyond the scope captured by ARIMA models, highlighting a limitation of traditional methods.

Compared to traditional methods, machine learning methods are more suitable for handling non-linear and non-stationary time series data. These methods typically involve feature engineering, selecting and constructing features related to birth rates such as age structure, education level, and economic development level. Then, regression analysis, decision trees, or support vector machines are used for modeling and prediction. These methods are advantageous in terms of being easy to interpret and implement, suitable for relatively simple data patterns. For example, Otoom et al. [11] compared 17 machine learning techniques in predicting population growth rates in the United Nations dataset, where random forest outperformed all other techniques in predictive performance and robustness to missing features. Huang et al. [39] used the LassoCV method and cluster analysis machine learning model to predict the birth rates of 31 provinces and cities in China based on birth rates and regional economic development levels. Zhang et al. [40] used various algorithms such as random forest, association rules, local variable importance, perceptual maps, and analogy analysis to explore the intrinsic relationships influencing the fertility levels of women of childbearing age and predict birth rates. However, traditional machine learning methods cannot accurately extract coupled features among complex multivariate variables, and due to the large number of parameters, their performance remains suboptimal.

With the rapid development of neural networks and computing resources, deep learning methods have been widely applied in recent years. These deep learning methods are capable of capturing complex nonlinear relationships within data, and they have shown good performance in tasks such as time series prediction of birth rates. Compared to traditional methods and machine learning, deep learning methods are better at handling long-term dependencies and nonlinear patterns among multiple variables or deep features, thereby improving prediction accuracy and robustness. Jia et al. [12] and Wu et al. [13] used BP neural networks to predict birth rates. Kumar et al. [41] proposed a deep learning model based on Convolutional Neural Network (CNN) and Recurrent Neural Network (RNN) for predicting birth rates in India, achieving excellent predictive results. Johann et al. [42] proposed a random CNN model for predicting birth rates and labor supply in Germany. However, traditional CNN-based deep learning methods have not effectively utilized temporal information among multiple variables and have not fully exploited the correlation within time series data, thus exhibiting certain limitations for tasks such as birth rate prediction.

### The studies on LSTM and attention mechanism

LSTM, a special network within the Recurrent Neural Network (RNN), is known for its unique gating structure and mechanism that enables it to capture long-term dependencies and handle non-linear patterns in time series data. This makes it suitable for complex multivariate time series forecasting tasks such as predicting birth rates. LSTM has been widely applied in various time series prediction tasks. Lu et al. [43] proposed a stock price prediction method based on CNN-LSTM, selecting eight input features including opening price, highest price, lowest price, closing price, trading volume, turnover, price fluctuation, and change. Kim et al. [44] presented a CNN-LSTM neural network that effectively extracts complex features of energy consumption for accurate residential energy consumption prediction. Guo et al. [45] utilized the LSTM method to model and detect the health status of bridges, enabling accurate prediction of potential anomalies in the future. Shahid et al. [46] introduced a bidirectional long short-term memory network (Bi-LSTM) for forecasting the COVID-19 pandemic. While these LSTM-based methods have shown promising results in their respective prediction tasks, traditional LSTM methods still face challenges in accurately extracting deep-seated clues and feature coupling relationships among complex multivariate time series data with specific objects, and may not better focus on variables with higher contributions.

Therefore, the industry has begun to attempt to combine attention mechanisms and LSTM methods to overcome the above-mentioned issues, and has further started to apply them in more complex multivariate time series data processing and prediction tasks. Lin et al. [47] proposed a double-stage encoder-decoder attention-based long short-term memory (LSTM) network for short-term regional load forecasting. Jiang et al. [48] combined LSTM with attention mechanisms and used TPE Bayesian optimization for hyperparameter optimization, achieving accurate indoor temperature prediction. Teng et al. [49] introduced a multi-scale local clue and hierarchical attention-based LSTM model (MLCA-LSTM) to capture potential stock price trend patterns, resulting in broader prediction coverage and more accurate results compared to the study [43]. Shi et al. [50] employ complementary attention to accurately predict and understand user intentions in social networks within cross-media multimodal data. Therefore, inspired by the above research, we first attempt to apply the advantages of combining attention mechanisms and LSTM to the prediction task of the birth rate in prefecture-level cities, proposing a BRP-Net dual-branch attention network model to extract deep temporal coupling features of specific prefecture-level city multivariate time series data, aiming to achieve accurate and robust birth rate prediction in prefecture-level cities.

## Method

### Overview

Recurrent neural network (RNN) is a machine learning algorithm used for processing time series data. Hochreiter et al. [51] proposed the long short-term memory (LSTM) model, which improved the generic RNN. The basic unit of the LSTM model is the memory cell, which includes four neural network layers: the cell state, forget gate, input gate, and output gate, instead of the simple tanh layer repeated in generic RNN. The LSTM model solves the problem of long-term dependencies that exist in the generic RNN. It can handle and predict important events with long time intervals and event sequence delays, and apply remote contextual information to the current moment. Therefore, using LSTM as the core model for predicting prefecture-level city birth rates can fully connect information data of the same region in different years, establish cross-scale feature connections, and achieve better prediction results. However, traditional LSTM models can only handle univariate time series data and often fail to effectively extract deep coupling clues and features among variables when handling multivariate time series data.

Therefore, we design a dual-branch attention structure based on LSTM. The advantage of this attention mechanism is that it overcomes the long-term dependency and information loss of RNN, better characterizes input data by assigning different importance to each element of the input network and focusing on more relevant inputs, and effectively learns the long-term temporal dependency and spatial correlation of complex multivariate time series. In practical terms, although economic development, education, healthcare, and population structure all have significant impacts on the birth rate, their respective influence weights are certainly different. By adaptively allocating different weights to each factor through the attention mechanism, it better aligns with practical significance and enhances the interpretability of the proposed network.

### The proposed network

**Fig 1:**
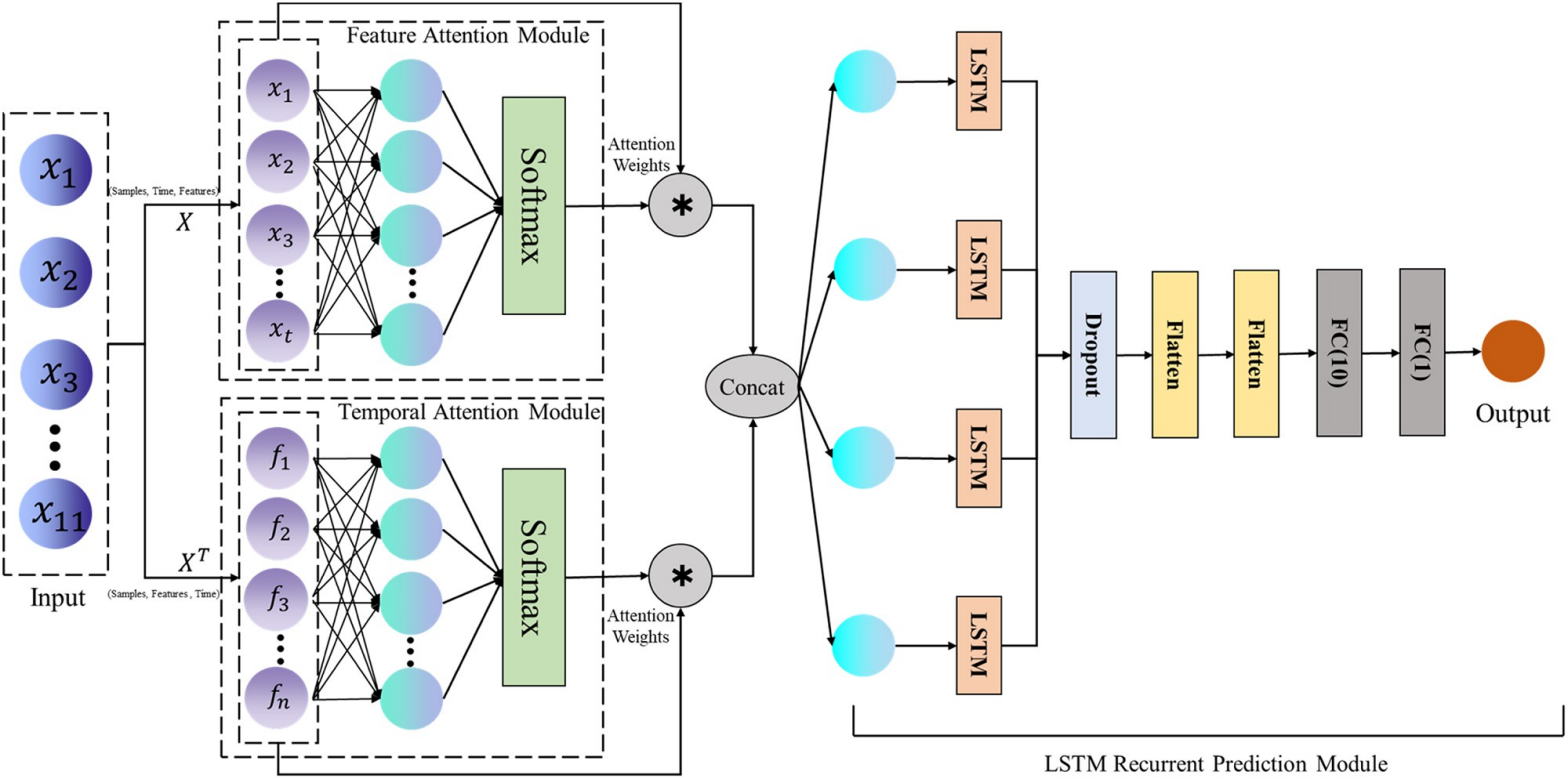
The structure of the BRP-Net proposed in this article.

The BRP-Net model we designed is a hybrid neural network consisting of three parts: the feature attention module, the temporal attention module, and the LSTM recurrent prediction module. Its complete structure diagram is shown in Fig 1. We selected 11 factors with the highest correlation with the birth rate in prefecture-level cities, such as house prices, the proportion of women of childbearing age, and the GDP level of prefecture-level cities, as the multidimensional input variables of the network model. The feature attention module learns the feature correlations in the multivariate input time series data. The temporal attention module and the feature attention module are in parallel structure, modeling the temporal relationships of multivariate input data based on transposed input data. The outputs of the two attention modules are generated by element-wise multiplication (denoted by *) of attention weights with input data, and the resulting output is combined and used as the input to the LSTM recurrent prediction module, ultimately outputting the predicted result value.

Inspired by the self-attention mechanism, we constructed the feature attention module and the temporal attention module using only input values and simplified single-layer perceptrons. Attention weights are applied to each input variable of each layer in the feature attention module and to each time step in the time attention module. The outputs *X*_*f*_ of the feature attention module and *X*_*t*_ of the temporal attention module are concatenated and used as the input to the LSTM model. The LSTM recurrent prediction module consists of a single-layer stateful LSTM and two fully connected layers. A stateful LSTM model means that the hidden state *h*_*t*_ learned at the current time step is transferred to the initial state in the next learning period. To prevent overfitting and ensure the robustness of the prediction results, we also applied a dropout layer after the output of the LSTM. After flattening, the output of the dropout layer is input to a fully connected layer for the final prediction output. Table 1 shows the hyperparameter settings used in each layer. To predict the final single true value, the number of neurons in the last fully connected layer is set to 1. The principles and details of the feature attention module, the temporal attention module, and the LSTM recurrent prediction module will be elaborated in detail in the following content.

**Table 1:** Model configurations.

Layer Name	Parameter Name	Value
LSTM	Unit size	6
	Activation function	Tanh
	Stateful	True
Dropout	Dropout rate	0.2
Fully connected	Number of neurons in the 1st FC layer	10
	Activation functions in the 1st FC layer	None
	Number of neurons in the 2nd FC layer	1
	Activation functions in the 2nd FC layer	None

#### The feature attention module

Inspired by the self-attention mechanism, we only use input values and a simplified single-layer perceptron to construct the feature attention module. The feature attention module can extract feature correlations among complex multivariate inputs and build a weighted feature representation to replace the original input. Specifically, given the value $x_{t}={\left(x_{t}^{1},x_{t}^{2},\ldots ,x_{t}^{n}\right)}^{T}\in R^{n}$ and the birth rate *y*_*t*_ of *n* input variables at time *t*, the attention weight $\alpha_{t}^{k}$ for the *k*-th input variable at time step *t* is calculated using Eqs (1) and (2). The softmax function is applied to *e*_*t*_ to ensure that the sum of all attention weights equals 1.

$$\begin{array}{c} e_{t}=W_{e}[x_{t};y_{t}]+b_{e} \end{array}$$ (1)

$$\begin{array}{c} \alpha_{t}^{k}=\frac{\text{exp}(e_{t}^{k})}{\sum_{i=1}^{n+1}\text{exp}(e_{t}^{i})} \end{array}$$ (2)

where $W_{e}\in R^{n+1\times n+1}$ and $b_{e}\in R^{n+1}$ are the trainable parameters. After calculating the attention weights for each time step, they are used to compute the average attention weight for the *k*-th input variable, as shown in Eq (3).

$$\begin{array}{c} \alpha^{k}=\frac{1}{T}\sum_{t=1}^{T}\alpha_{t}^{k} \end{array}$$ (3)

Finally, we calculate the weighted input variable values *X*_*f*_ by applying attention weights to each input variable.

$$\begin{array}{c} X_{f}=(x^{1}\alpha^{1}\cdot ,x^{2}\alpha^{2},\ldots ,x^{n}\alpha^{n},y\alpha^{n+1}) \end{array}$$ (4)

The output *X*_*f*_ of the feature attention module, where $X_{f}\in R^{T\times (n+1)}$, is combined with the output of the temporal attention module through concatenation and input into the LSTM recurrent prediction layer.

#### The temporal attention module

Just like the number of features, the time scale of historical data on population development is also an important factor affecting the performance of time series regression. In order to identify the key time points of historical data for the same prefecture-level city in different years, we designed a parallel temporal attention module. While the feature attention module applies attention weights to each input variable differently, the temporal attention module applies temporal attention weights at each time step. Given the time series data of the *k*-th input variable $x^{k}={\left(x_{1}^{k},x_{2}^{k},\ldots ,x_{T}^{k}\right)}^{T}\in R^{T}$, the temporal attention mechanism is implemented in a similar way to the feature attention mechanism. The difference lies in that the temporal attention weights are calculated based on the time axis. They are calculated by Eqs (5) and (6) as follows:

$$\begin{array}{c} e^{k}=W_{t}x^{k}+b_{t} \end{array}$$ (5)

$$\begin{array}{c} \alpha_{t}^{k}=\frac{\text{exp}(e_{t}^{k})}{\sum_{i=1}^{n+1}\text{exp}(e_{t}^{k})} \end{array}$$ (6)

where $W_{t}\in R^{T\times T}$ and $b_{T}\in R^{T}$ are the trainable parameters. In addition to the input variables, the temporal attention module is also applied to predict the population birth rate data *y* before the prediction time. We utilize weights when calculating the attention weights for input variables.

$$\begin{array}{c} e^{y}=W_{t}y+b_{t} \end{array}$$ (7)

$$\begin{array}{c} \alpha_{t}^{y}=\frac{\text{exp}(e_{t}^{y})}{\sum_{i=1}^{n+1}\text{exp}(e_{t}^{y})} \end{array}$$ (8)

When estimating the attention weights for each input variable and historical population birth rate data, the variables are used to calculate the average attention weight at time step *t*, as shown in Eq (9), where $\alpha^{l}\in \left(\alpha^{1},\alpha^{2},\alpha^{n},\alpha^{y}\right)$.

$$\begin{array}{c} \alpha^{l}=\frac{1}{T}\sum_{t=1}^{T}\alpha_{t}^{l} \end{array}$$ (9)

At each time step, attention weights can be applied to compute the input variable *X*_*t*_, with the input variable *X*_*t*_ being weighted at each time step.

$$\begin{array}{c} X_{t}=(x^{1}\alpha^{1}\cdot ,x^{2}\alpha^{2},\ldots ,x^{n}\alpha^{n},y\alpha^{y}) \end{array}$$ (10)

The output $X_{t}\in R^{T\times (n+1)}$ of the temporal attention module and the output of the feature attention module are combined through concatenation and then input into the LSTM recurrent prediction layer.

#### The LSTM recurrent prediction module

**Fig 2:**
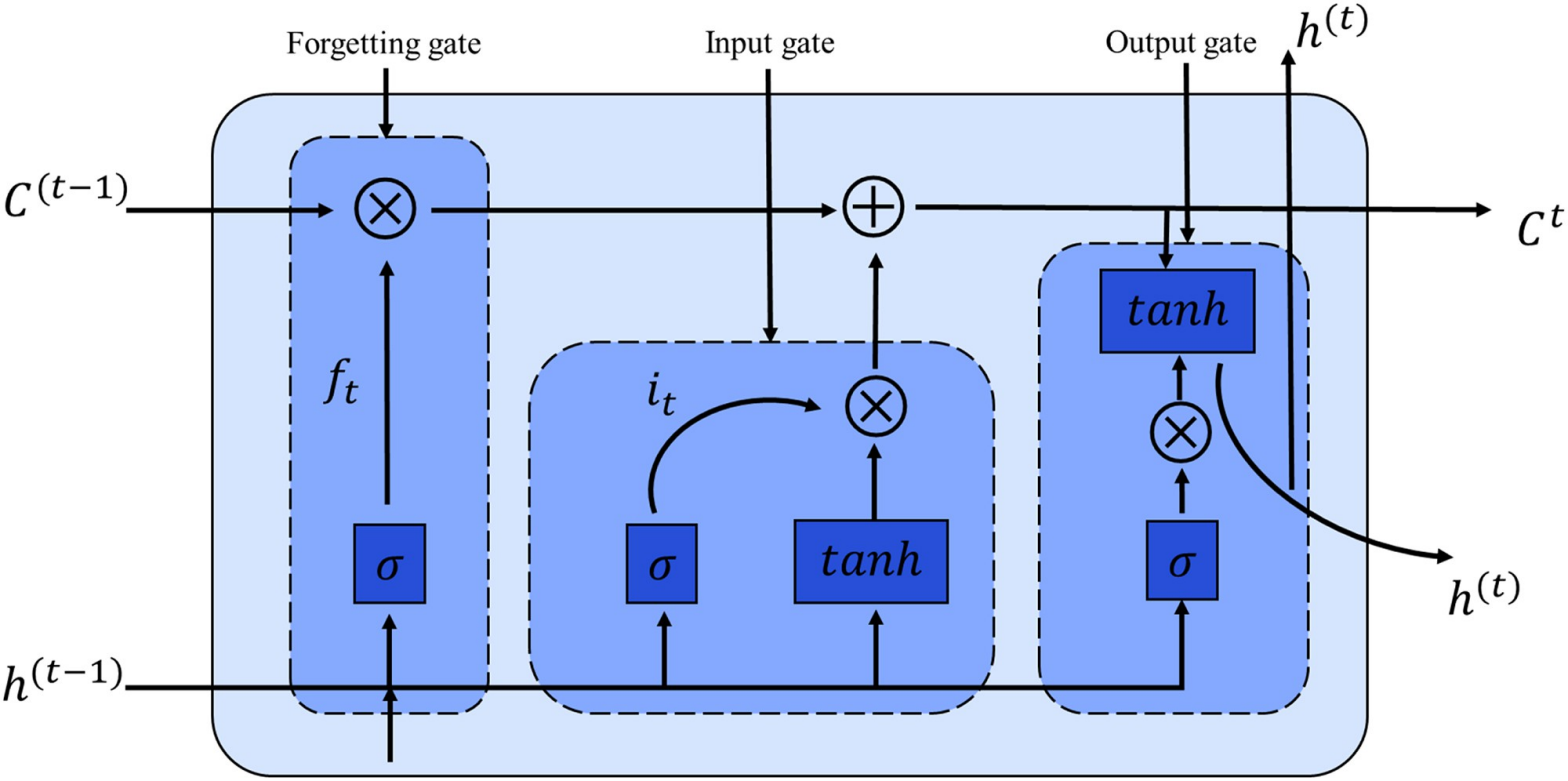
The structure of the LSTM.

The LSTM architecture used in this article is shown in Fig 2. An LSTM unit consists of three gate units. Long short-term memory (LSTM) is a well-known deep learning architecture used for processing time series data. It incorporates gate mechanisms and internal states to enable selective accumulation of historical information and retention of new information [52]. Fig 2 illustrates the structure of an LSTM unit. The LSTM unit comprises three gates, namely the forget gate *f*_*t*_, input gate *i*_*t*_, and output gate *o*_*t*_. The forget gate *f*_*t*_ decides how much historical information to retain in the internal state when new information is introduced. The input gate *i*_*t*_ determines which new information should be updated to the internal state, while the output gate *o*_*t*_ selects which updated information can be passed as input to the network for the next time step [7]. When dealing with time series prediction problems, the LSTM network has faster convergence speed and higher accuracy compared to other neural network models. The following equation represents the internal operation of the LSTM unit:

$$\begin{array}{c} \left\{ \begin{array}{cc} i_{t} & =\sigma (W_{i}\cdot [h_{t-1},x_{t}]+b_{i})\\ f_{t} & =\sigma (W_{f}\cdot [h_{t-1},x_{t}]+b_{f})\\ o_{t} & =\sigma (W_{o}\cdot [h_{t-1},x_{t}]+b_{o})\\ c_{t}^{\prime } & =\text{tanh}(W_{c}\cdot [h_{t-1},x_{t}]+b_{c})\\ c_{t} & =f_{t}⊙c_{t-1}+i_{t}⊙c_{t}^{\prime }\\ & h_{t}=o_{t}⊙\text{tanh}(c_{t}) \end{array}\right. \end{array}$$ (11)

here, *h* is the hidden state and x is the input variable. $c_{t}^{\prime }$ represents the candidate memory information. *W* denotes the weight of the neural network and *b* denotes the bias of the neural network.

### Loss function and evaluation index

To further optimize the model parameters, it is necessary to minimize the reconstruction error. We have used mean squared error as the loss function, which is expressed as follows:

$$\begin{array}{c} L\left(x,z\right)=\frac{1}{n}\sum_{i=1}^{n}{\left(x_{i}-{\hat{x}}_{i}\right)}^{2} \end{array}$$ (12)

where ${\hat{x}}_{i}$ represents the predicted value of the prefecture-level city’s birth rate, and *x*_*i*_ represents the true value.

In this article, we have evaluated the predictive performance of the model using three evaluation metrics: root mean squared error (RMSE), mean relative error (MRE) and coefficient of determination (R-squared score).

$$\begin{array}{c} RMSE=\sqrt{\frac{1}{n}\sum_{i=1}^{n}{\left(|y_{i}-{\hat{y}}_{i}|\right)}^{2}} \end{array}$$ (13)

$$\begin{array}{c} MRE=\frac{1}{N}\sum_{i=1}^{n}\frac{\left|y_{i}-{\hat{y}}_{i}\right|}{y_{i}}\times 100\% \end{array}$$ (14)

$$\begin{array}{c} R^{2}=1-\frac{\sum_{i=1}^{n}{\left(y_{i}-{\hat{y}}_{i}\right)}^{2}}{\sum_{i=1}^{n}{\left(y_{i}-\bar{y}\right)}^{2}} \end{array}$$ (15)

where ${\hat{y}}_{i}$ represents the predicted value, *y*_*i*_ represents the real value, and n represents the number of data points. A smaller RMSE value indicates a smaller prediction error of the prefecture-level city’s birth rate prediction model. The closer the R-squared value is to 1, the better the fit between the predicted prefecture-level city’s birth rate and the actual value.

### Design of indicators for policy-based birth quota impact

As the strict family planning policy has encountered resistance in some regions, the government allows each province to formulate different birth policies based on their population status to ensure policy implementation. To control the normative impact of different birth policies in different regions, this article proposes a method for calculating the Policy-Based Birth Quota (PBQ) inspired by Guo (2003). We calculate the PBQ allowed for each family under the “one-child policy” based on different family types and ethnic structures, with specific variables shown in Table 2. We classify China’s prefecture-level cities into three categories by province: the first category includes Beijing, Tianjin, Jiangsu, Chongqing, and Sichuan, where the “one-child policy” is widely implemented. The second category includes Jilin, Liaoning, Hebei, Shanxi, Shaanxi, Gansu, Shandong, Zhejiang, Henan, Anhui, Hubei, Hunan, Fujian, and Guangdong, where the proportion of ethnic minorities is relatively small, and the PBQ only considers regional differences. The calculation formula is as follows:

$$\begin{array}{c} PBQ=(I\times A+1\times B)\times J \end{array}$$ (16)

**Table 2:** Explanations of factors represented in the calculation process of PBQ.

Serial number	Representative meaning
A	Proportion of agricultural population(which can have 2 children if their first child was female)
B	Proportion of non-agricultural population
C	Proportion of Han population
D	Proportion of ethnic minority population(some provinces have different policies for different minorities)
E	Proportion of ethnic minority population under 10 million(which can have 2children)
F	Proportion of ethnic minority population not under 10 million
G	Proportion of Tibetan population
H	Proportion of non-Tibetan population
I	Gender ratio at birth
J	Adjustment coefficient for special circumstances of childbirth

The third category includes Inner Mongolia, Tibet, Xinjiang, Qinghai, Ningxia, Guizhou, Yunnan, Guangxi, and Hainan, where the population has different structures. The calculation formulas are as follows:

$$\begin{array}{c} PBQ=(2\times E+I\times A\times F+1\times B\times F)\times J \end{array}$$ (17)

$$\begin{array}{c} PBQ=(2\times A\times D+I\times A\times C+1\times B)\times J \end{array}$$ (18)

$$\begin{array}{c} PBQ=(2\times A+1\times B)\times J \end{array}$$ (19)

$$\begin{array}{c} PBQ=(2\times (1-B\times C)+1\times B\times C))\times J \end{array}$$ (20)

$$\begin{array}{c} PBQ=(3\times A\times D+2\times B+2\times A\times C+1\times B\times C)\times J \end{array}$$ (21)

$$\begin{array}{c} PBQ=(2\times (1-B\times F)+1\times B\times F)\times j \end{array}$$ (22)

$$\begin{array}{c} PBQ=(2\times A+2\times B\times D+1\times B\times C)\times J \end{array}$$ (23)

$$\begin{array}{c} PBQ=(3\times G\times A+2\times G\times B+1)\times (1-G) \end{array}$$ (24)

Eq 17 represents Inner Mongolia and Guangxi, while Eqs 18–24 represent Guizhou, Yunnan, Ningxia, Xinjiang, Hainan, Qinghai, and Tibet, respectively.

It is worth noting that after the second-child policy was implemented in 2016, the PBQ value for all prefecture-level cities was 2. The introduction of the PBQ indicator can more effectively measure the impact of local government fertility policies on the birth rate, making the overall prediction results more accurate and interpretable. The results of the ablation experiments in the next section also confirm the effectiveness of the proposed PBQ indicator.

## Experiment and evaluation

### Dataset

To validate the effectiveness of the BRP-Net method in predicting the birth rate of prefecture-level cities, we collected comprehensive data from 361 prefecture-level cities in China from 2000 to 2020, forming a widely covered and regionally diverse dataset of birth rates. The birth rate data for 2010 and 2020 are derived from nationwide population census data, and the data for 2005 and 2015 are from 1% population sample surveys. All the data were collected and compiled from the statistical bulletins of the National Bureau of Statistics and various prefecture-level cities. Data for other years are sourced from the provincial statistical bureaus to which the prefecture-level cities belong.

Table 3 presents the birth rate situation from 2000 to 2020. From the content of the above figures and tables, it can be seen that there is significant heterogeneity in the fluctuation of birth rates among different prefecture-level cities in China. For example, in 2000, the lowest birth rate in Haikou City was only 3.77‰, while the highest in Wenzhou City reached 27.25‰, resulting in a difference of 23.48‰. By 2019, the birth rate in Baicheng City, Jilin Province, was only 3.4‰, while in Shenzhen City, it reached 21.68‰, resulting in a range of 24.34‰. These significant differences are caused by various reasons such as local economic development and educational levels.

**Table 3:** The birth rate situation of prefecture-level cities published from 2000 to 2020.

Year	Sample Size	Mean	Maximum	Minimum	Range	Standard Deviation
2000	294	12.39	27.25	3.77	23.48	3.73
2001	238	10.72	20.87	3.12	17.75	3.53
2002	241	10.41	20.37	3.03	17.34	3.29
2003	243	10.08	21.10	3.31	17.79	3.28
2004	238	10.76	18.83	3.21	15.62	2.79
2005	292	10.81	20.61	5.88	14.73	2.74
2006	270	10.48	18.30	3.13	15.17	2.52
2007	260	10.70	17.39	6.00	11.39	2.38
2008	261	10.81	24.31	5.60	18.71	2.70
2009	262	10.75	20.85	5.50	15.35	2.51
2010	290	10.85	20.23	2.91	17.32	2.77
2011	243	10.63	18.23	4.96	13.27	2.51
2012	257	11.15	26.01	5.21	20.8	2.57
2013	252	11.00	19.40	5.12	14.28	2.45
2014	259	11.42	22.00	4.87	17.13	2.50
2015	259	11.08	24.98	4.21	20.77	2.97
2016	260	11.60	18.10	4.74	13.36	2.68
2017	263	12.28	25.07	4.40	20.67	3.12
2018	260	11.08	18.70	3.84	14.86	2.83
2019	288	10.25	21.68	3.40	18.28	2.83
2020	289	11.65	20.37	3.43	22.67	2.39

Hence, it can be seen that there are numerous and extensive factors that affect the birth rate of prefecture-level cities, and they are interrelated to a certain extent. Therefore, in selecting the input variables for the network, we have chosen twelve factors based on their significant impact on the birth rate of prefecture-level cities. These factors include GDP, PBQ, registered population relative to de jure population, primary school teachers per 10,000 people, number of practitioners per 10,000 people, number of university students per 10,000 people, old-age dependency ratio, per capita social security and employment expenditure, housing price relative to GDP, female education level, and percentage of employed women. These factors essentially cover various aspects of a prefecture-level city, such as economic development, educational level, and age-appropriate population structure. The detailed descriptive statistical data for these factors are shown in Table 4.

**Table 4:** Descriptive statistics of main variables.

Variables	Samples	Mean	Maximum	Minimum	Standard error
birthrate(‰)	4309	9.54	19.46	1.70	2.73
GDP	4309	41,835	180,044	2,396	32,331
PBQ	4309	1.56	2.03	1.06	0.32
registered pop relative to de jure pop	4309	0.02	3.23	-0.48	0.28
primary school teachers	4309	43.05	121.60	19.12	9.99
practitioners	4309	20.86	75.19	5.49	8.45
college students	4309	168.00	1294.00	4.58	218.20
old-age dependency ratio (%)	4309	0.17	0.48	0.02	0.08
social security and employment expenditure	4309	856.40	7265	4.11	762.10
housing price relative to GDP	4309	0.13	0.39	0.02	0.06
Percentage of women with a university degree or above (%)	4309	0.09	0.42	0.01	0.07
percentage of employed women (%)	4309	0.44	0.63	0.10	0.04

### Implement details

We partitioned the data into training, validation, and test sets in an 8:1:1 ratio. Adam [8] was utilized as the optimization algorithm, and the network was implemented in the Pytorch [9] framework. For testing, a system with one Core i9–12900KF CPU, one NVIDIA RTX3090ti GPU, and 64GB of memory was used. We used a batch size of 32 and a learning rate of 0.001. Our model was trained for a total of 1000 epochs, taking a total of 156 minutes.

### Experimental results and comparative analysis of birth rate prediction in prefecture-level cities

To validate the predictive accuracy of the proposed BRP-Net model, we established several benchmark models and compared our model’s performance with other similar models. These benchmark models include classic traditional methods such as ARIMA, VAR, as well as learning-based methods like CAE, LSTM, GRU, CNN-LSTM:

**ARIMA**: Based on the autocorrelation and moving average properties of time series, it models data through differencing, autoregression, and moving averages. The ARIMA model can identify trends and seasonality in the data for prediction.

**VAR**: A multivariate time series modeling method that considers the mutual influence between variables. It involves selecting appropriate lag orders, estimating VAR model parameters, and making predictions.

**CAE**: Convolutional Autoencoder. It utilizes convolutional neural networks for feature extraction and compression, followed by decoder reconstruction of the input.

**LSTM**: A type of recurrent neural network structure capable of capturing long-term dependencies. It takes in time series input, learns long-term dependencies, and outputs predictions.

**GRU**: Similar to LSTM but with a simpler structure, featuring update and reset gates. It learns inherent patterns and dependencies in time series data for prediction.

**CNN-LSTM**: Combines the feature extraction capability of CNN with the time series modeling ability of LSTM. This model first extracts features through CNN and then inputs them to LSTM for sequence modeling and prediction.

**GCN-LSTM**: The GCN-LSTM model is based on the T-GCN model structure proposed by Zhao et al. [53]. T-GCN combines graph convolutional layers [54] and the GRU model, and has been applied to time series data prediction tasks such as electricity load forecasting and traffic flow prediction.

**DA-RNN**: The DA-RNN model proposed by Qin et al. [55] is an encoder-decoder structured model with attention mechanisms applied in both the encoder and decoder. This model has demonstrated outstanding performance in indoor temperature forecasting and exchange rate prediction, covering relatively long time spans.

**Fig 3:**
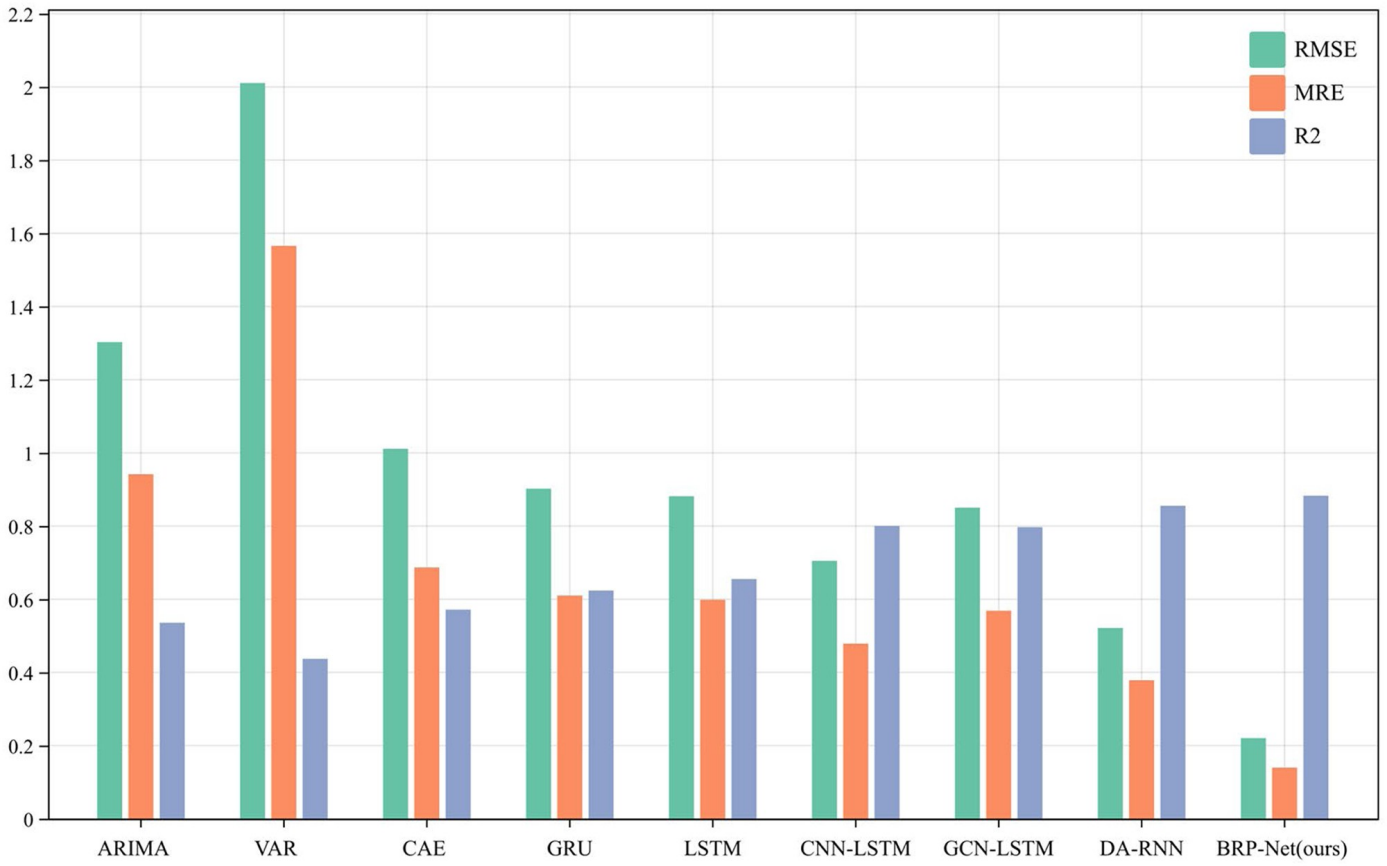
Visualization of the compared methods on RMSE, MRE and *R*^2^.

In addition to traditional methods, all learning-based methods used for comparison underwent training for 1000 epochs to reduce random errors. We utilized root mean square error (RMSE), mean relative error (MRE), and coefficient of determination (R-squared score) to compare results. The results of the quantitative experiments and their visualization are shown in Table 5 and Fig 3. The experimental results indicate that overall, the prediction errors of traditional methods are higher than those of learning-based methods. Among the learning-based methods, CAE exhibits the highest average MRE and RMSE at 75.5%, possibly due to its weaker learning capability compared to other benchmark models and its inability to capture temporal relationships in multivariate input data. Subsequently, LSTM and GRU recorded the second and third highest error rates, with MRE and RMSE at 0.5986, 0.6100 and 0.8815, 0.9021, respectively, possibly due to the inability of pure LSTM and GRU to cover complex and long-span multi-input variables. Relatively, although GCN-LSTM’s performance is slightly better than LSTM and GRU, it still falls short of models like CNN-LSTM, which first extracts multivariate features before conducting time series prediction. This may be attributed to the limitations of graph convolutional layers optimized for learning spatial information in capturing strong time series features of birth rates. Encoders with input attention mechanisms and decoders with temporal attention mechanisms in DA-RNN and the proposed BRP-Net exhibit excellent performance. The reason for these results is that both models use attention mechanisms to effectively learn time series features of input data and input variable features. The BRP-Net model records the smallest error results in terms of RMSE and MRE at 0.2206 and 0.1397, respectively, with R-squared closer to 1 compared to other models, indicating robustness and higher predictive accuracy. The predicted birth rates of Chinese prefecture-level cities are also closer to the actual values with smaller errors.

**Table 5:** Qualitative comparison of birth rate prediction results in prefecture-level cities. Bold is best.

Method	RMSE	MRE	*R* ^2^
ARIMA	1.3027	0.9414	0.5357
VAR	2.0108	1.5657	0.4372
CAE	1.0112	0.6866	0.5716
GRU	0.9021	0.6100	0.6237
LSTM	0.8815	0.5986	0.6553
CNN-LSTM	0.7049	0.4786	0.8002
GCN-LSTM [53]	0.8502	0.5686	0.7970
DA-RNN [55]	0.5215	0.3786	0.8553
BRP-Net(ours)	**0.2206**	**0.1397**	**0.8829**

**Fig 4:**
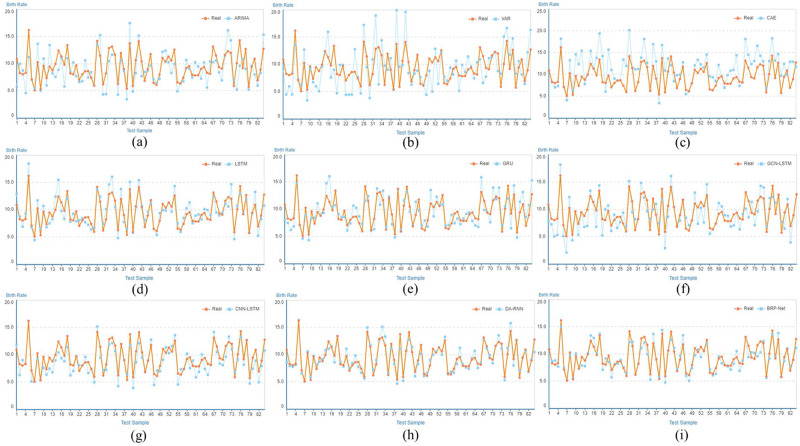
Visualization and comparison results with traditional and learning-based methods for birth rate prediction in prefecture-level cities. (a)ARIMA, (b)VAR, (c)CAE, (d)LSTM, (e)GRU, (f)GCN-LSTM [53], (g)CNN-LSTM, (h)DA-RNN [55], (i)BRP-Net(ours).

Fig 4 displays the visual results of population birth rate predictions for each model at the prefecture level. From these qualitative experiments, it is evident that traditional methods exhibit significant fluctuations. In contrast, our proposed BRP-Net method achieves precise and robust prediction results.

### Ablation experiment

**Fig 5:**
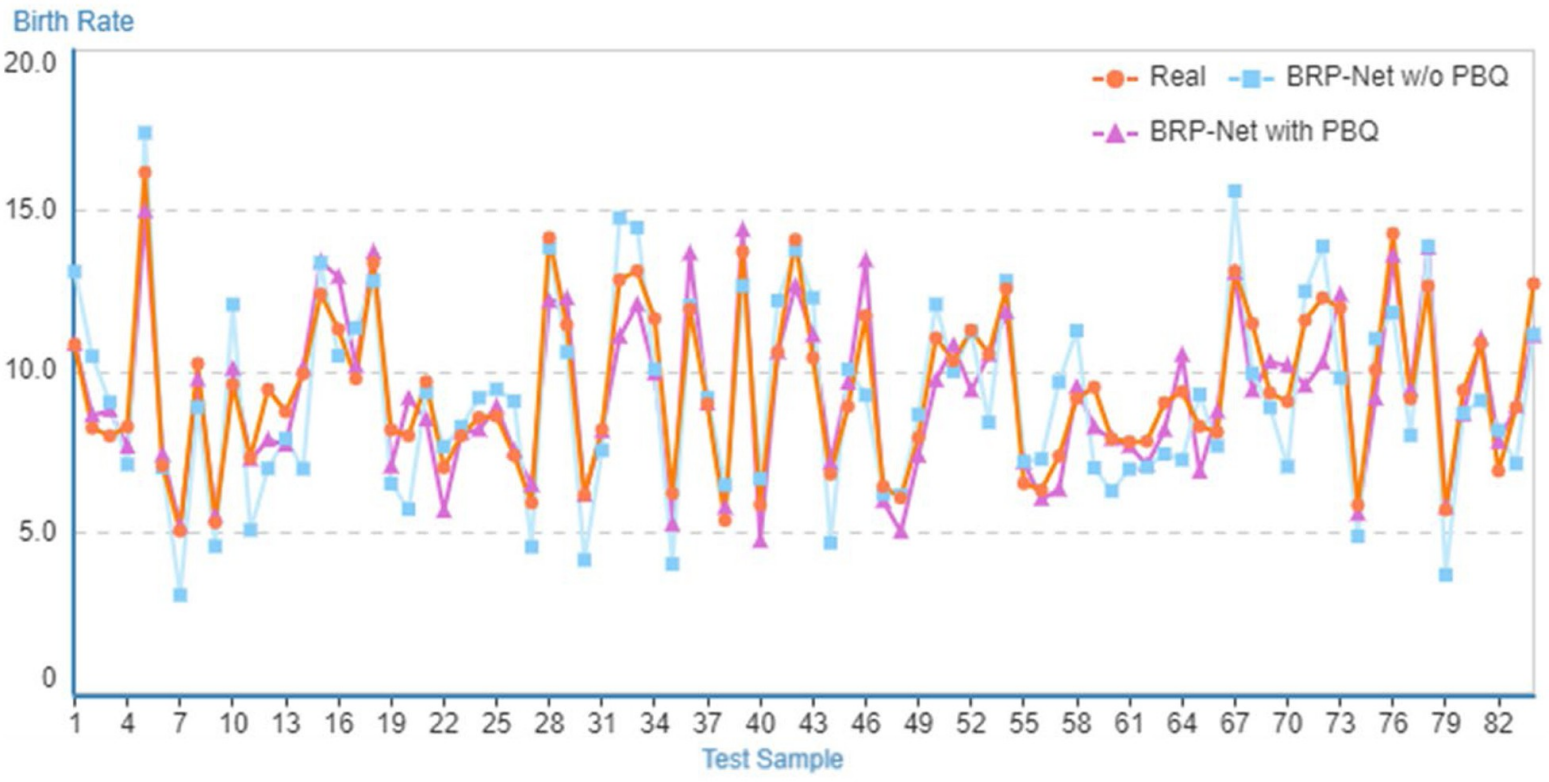
Visualization of comparative results for ablation experiments targeting PBQ metrics.

To measure the effectiveness of the PBQ index proposed in this paper, we conducted ablation experiments on this index. The BRP-Net without the PBQ index reduced from eleven input variables to ten. The results of the ablation qualitative and quantitative experiments are presented in Table 6 and Fig 5. These results clearly demonstrate that excluding the PBQ as an input variable for prefecture-level population birth rate prediction leads to a certain degree of reduction in prediction accuracy. This also indicates that incorporating local policies into the influencing factors of prefecture-level population birth rates, as proposed in the BRP-Net, aligns with the actual situation in China, thus achieving more accurate prediction accuracy under the same model.

**Table 6:** Quantitative ablation experiments to measure the effectiveness of the PBQ index. Bold is best.

Method	RMSE	MRE	*R* ^2^
BRP-Net w/o PBQ	0.3915	0.2571	0.8133
BRP-Net with PBQ	**0.2206**	**0.1397**	**0.8829**

### Correlation analysis and contribution weight analysis of factors affecting birth rate

**Fig 6:**
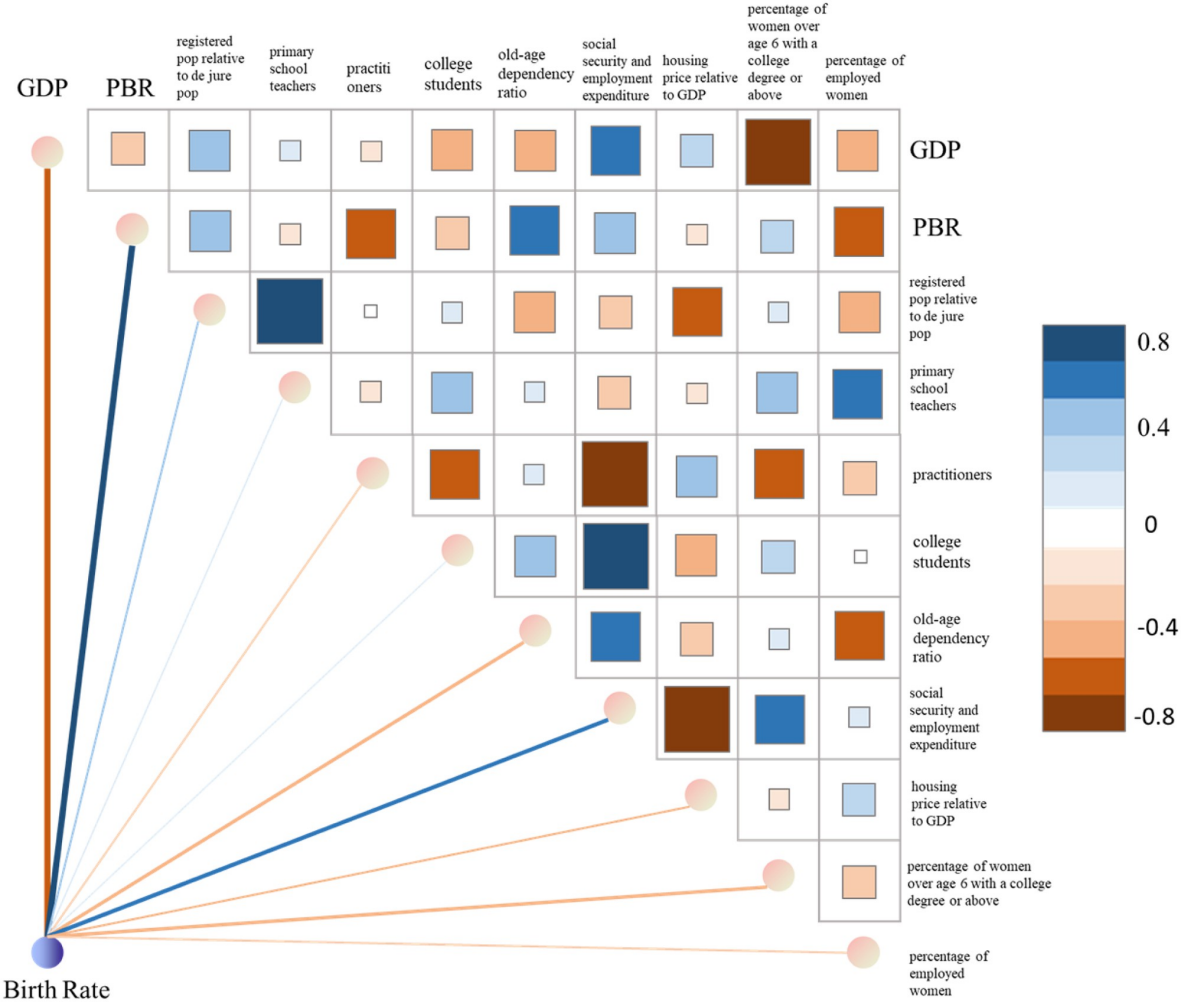
Heatmap of correlation among multi-dimensional input variables and importance assessment plot.

We also conducted correlation analysis on the attribute features of the input multivariate data to provide support for prefecture-level city governments to comprehensively control local development direction and improve birth rates. The heatmap represents the size of the correlation coefficient, and the depth of color represents the strength of the correlation. In Fig 6, the relationship between color and correlation strength corresponds to the color bar on the right side of the figure. The more blue it is, the stronger the positive correlation, and the more orange it is, the stronger the negative correlation. By analyzing the correlation results of the heatmap, it can be found that the overall linear correlation between variables is not strong, which is a key premise to ensure that the proposed model can fully explore the time series feature information of different variables.

At the same time, we evaluated the feature importance of the predicted results of the proposed BRP-Net through SHapley Additive exPlanations (SHAP) [56]. This method mainly obtains the feature importance evaluation by iteratively calculating feature interactions, differential feature calculation, and weighted average calculation of Shapley values for all feature subsets. This theory provides a global explanation method for model prediction, which can help understand the degree of influence of each input feature on the prediction results in neural networks. The experimental results are also shown in Fig 6. Similarly, the thickness of the line represents the size of the correlation coefficient, and the depth of color represents the strength of the correlation. The more blue it is, the stronger the positive correlation, and the more orange it is, the stronger the negative correlation. We can see that GDP and PBQ have the most significant impact on birth rate in prefecture-level cities, with the former showing a negative correlation connection and the latter showing a positive correlation connection. In addition, social security and employment expenditure, percentage of women over age 6 with a college degree or above, and old-age dependency ratio also have a significant impact on birth rate in prefecture-level cities.

## Conclusion

In this paper, we established a BRP-Net model based on the multidimensional time series data of comprehensive population development in China’s prefecture-level cities for predicting population birth rates. This model, built upon LSTM and incorporating attention mechanisms, features a dual-branch attention structure comprising feature attention modules and temporal attention modules. It better captures and extracts complex spatial feature correlations and cross-temporal scale dependencies of prefecture-level cities population development multidimensional variables, enabling accurate prediction of birth rates at the prefecture level. Experimental results demonstrate that the proposed BRP-Net model outperforms traditional models and other learning-based models in terms of fitting and prediction accuracy, robustness, and generalization performance. Additionally, we further analyzed the spatial linear correlations of the aforementioned multidimensional variables and their importance for birth rate prediction, providing visualized experimental results. Moreover, this study holds profound social significance. Against the backdrop of declining birth rates, from a national strategic perspective, this research can provide decision-making support for prefecture-level city governments to formulate more accurate and rational fertility encouragement policies. Prefecture-level city governments can utilize comprehensive development forecast data of cities combined with our proposed BRP-Net model to predict future birth rates and adjust corresponding urban development policies based on the predicted results.

The main focus of future research will be to further explore the prediction of birth rates in prefecture-level cities. On the one hand, we plan to investigate the differences in birth rates and their influencing factors among Chinese prefecture-level cities based on this study. Furthermore, we aim to apply the proposed BRP-Net model to city predictions in other countries, analyzing the impact of different national conditions and policies on birth rates in prefecture-level cities. On the other hand, continuous model improvement can be achieved by utilizing more advanced networks and modules to enhance the accuracy and generalization capabilities of the model.

## Supporting information

S1 File All figures in this article. (PDF)

S2 File Raw comprehensive data on prefecture-level city development used for validation and testing in this article. (ZIP)

S3 File The code for the implementation of BRP-Net proposed in this paper. (ZIP)
