# Supplementary material for: BRP-Net: A discrete-aware network based on attention mechanisms and LSTM for birth rate prediction in prefecture-level cities
Source: PLoS One. 2024 Sep 12;19(9):e0307721. doi: 10.1371/journal.pone.0307721 (PMC11392232; doi:10.1371/journal.pone.0307721)
Supplement: S1 File — (PDF) [file pone.0307721.s001.pdf]

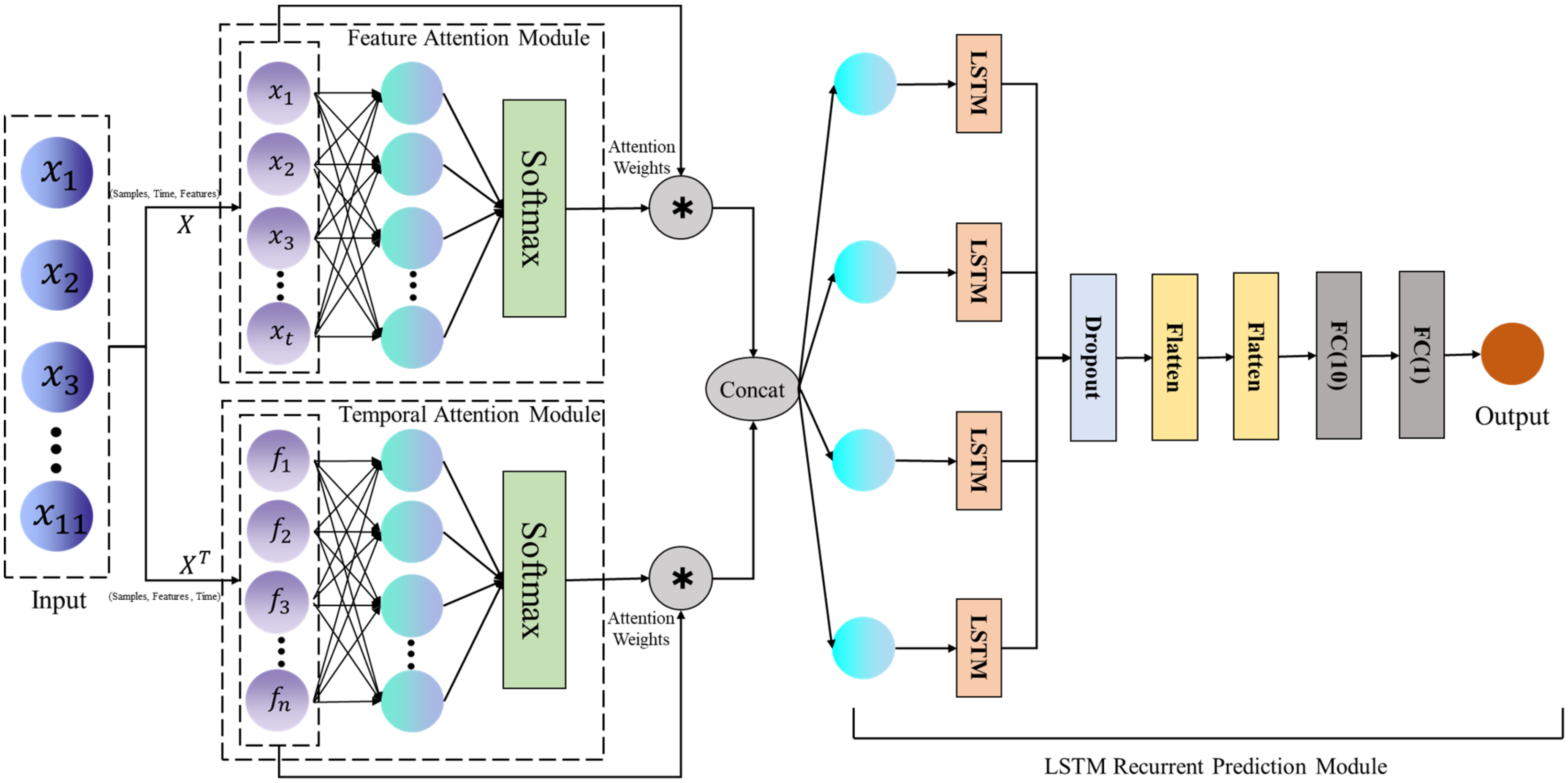


Fig 1. The structure of the BRP-Net proposed in this article.

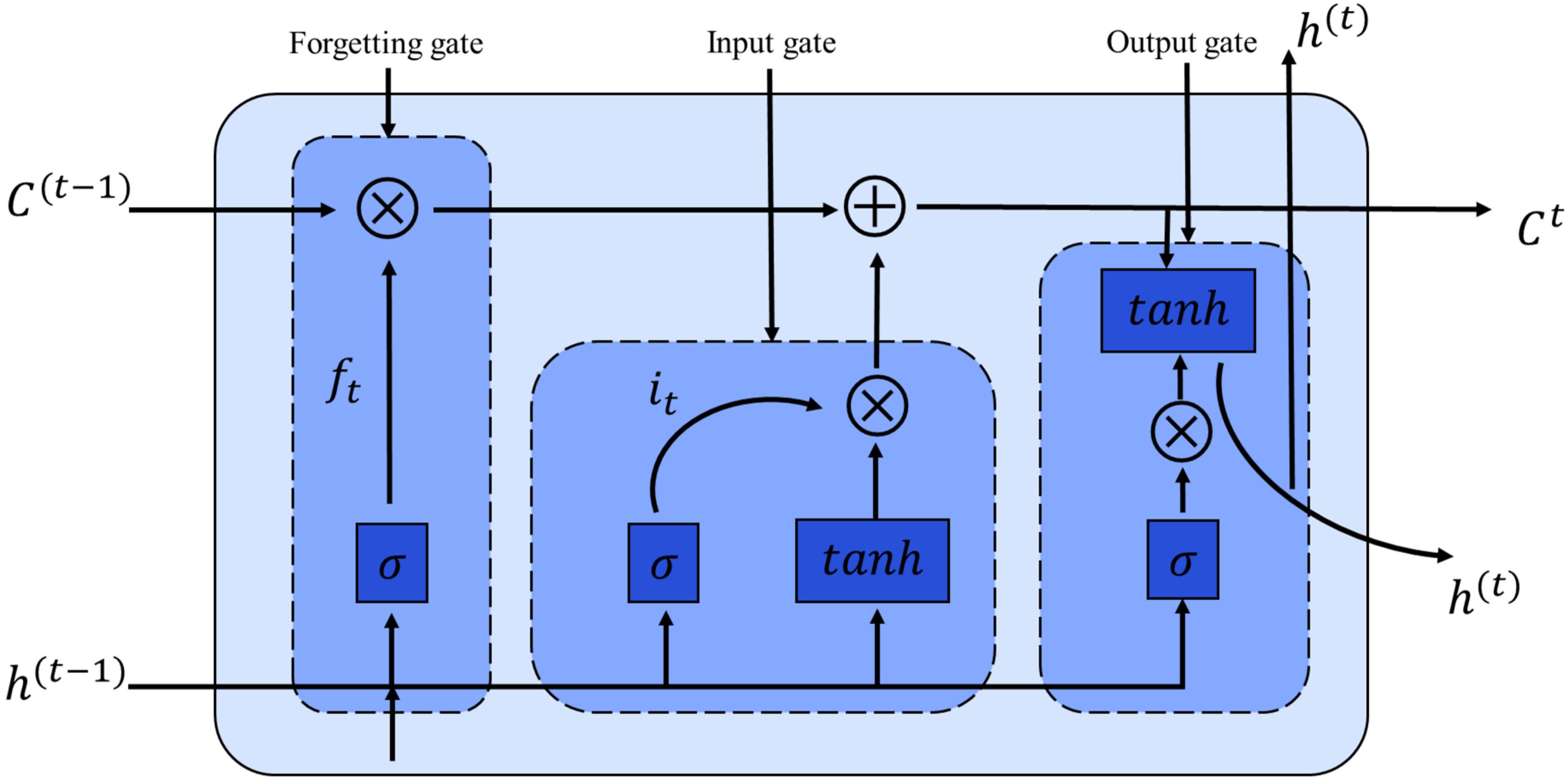


Fig 2. The structure of the LSTM.

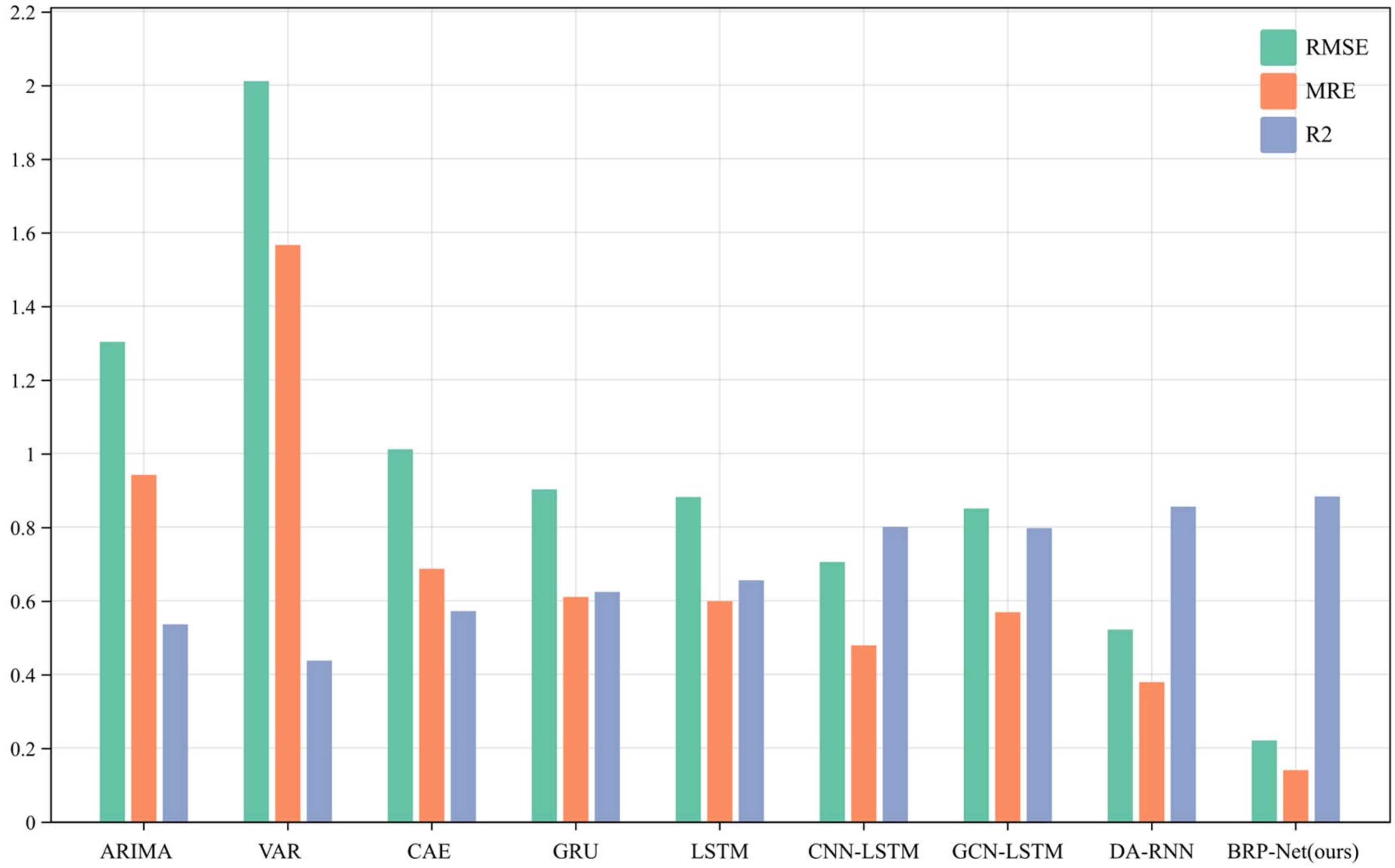


Fig 3. Visualization of the compared methods on RMSE, MRE and $R^2$.

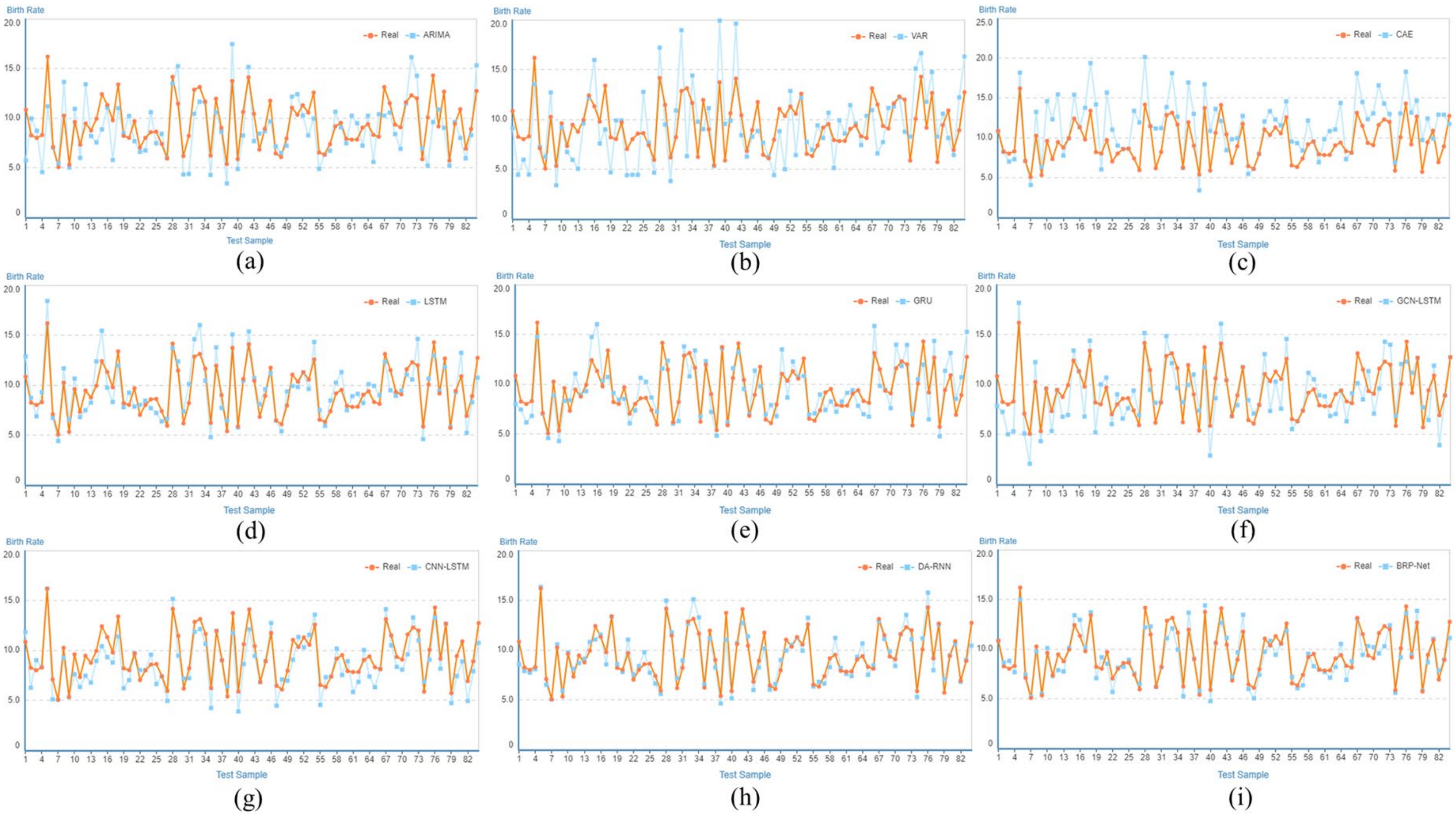


Fig 4. Visualization and comparison results with traditional and learning-based methods for birth rate prediction in prefecture-level cities. (a)ARIMA, (b)VAR, (c)CAE, (d)LSTM, (e)GRU, (f)GCN-LSTM [55], (g)CNNLSTM, (h)DA-RNN [57], (i)BRP-Net(ours).

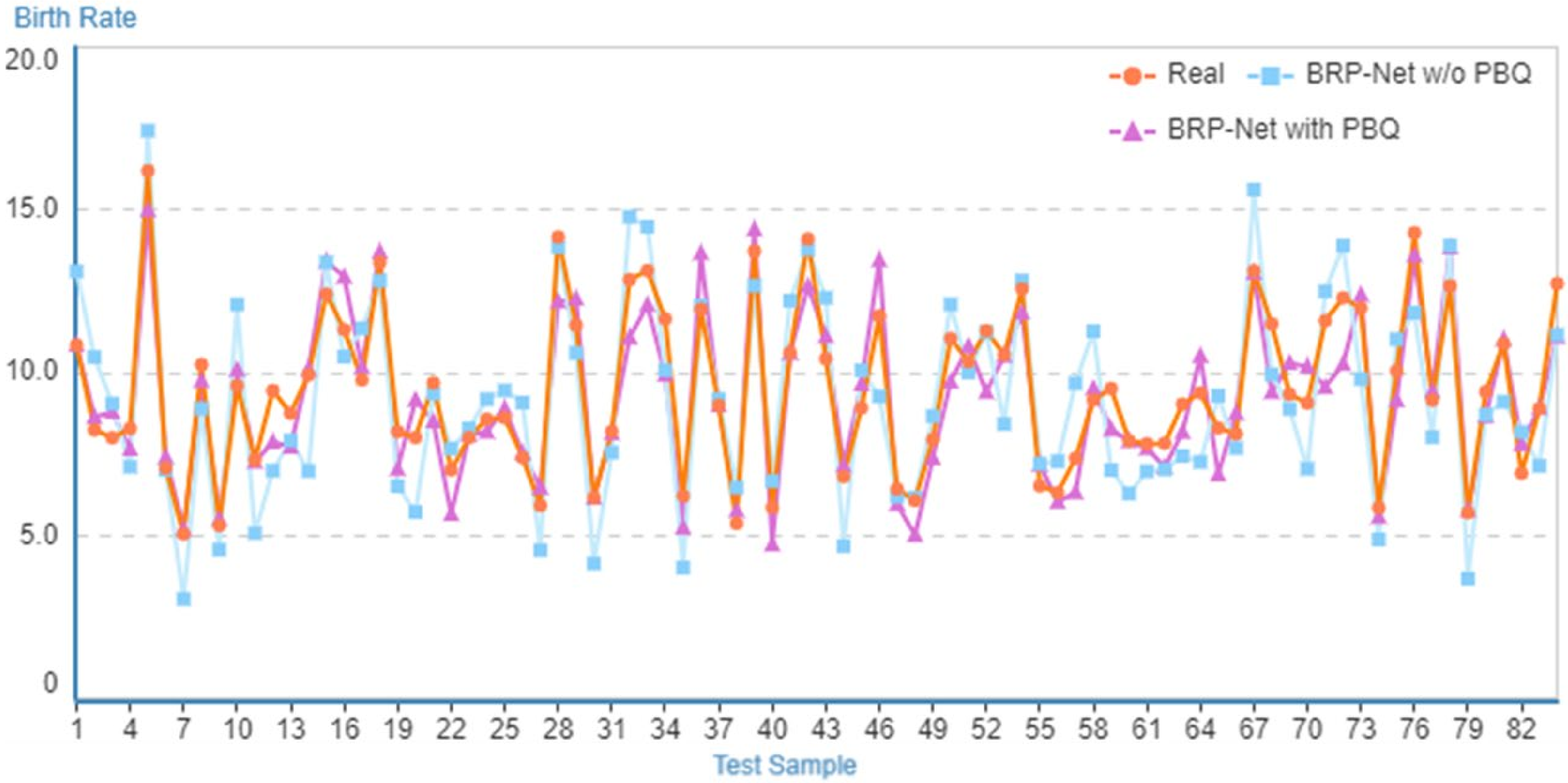


Fig 5. Visualization of comparative results for ablation experiments targeting PBQ metrics.

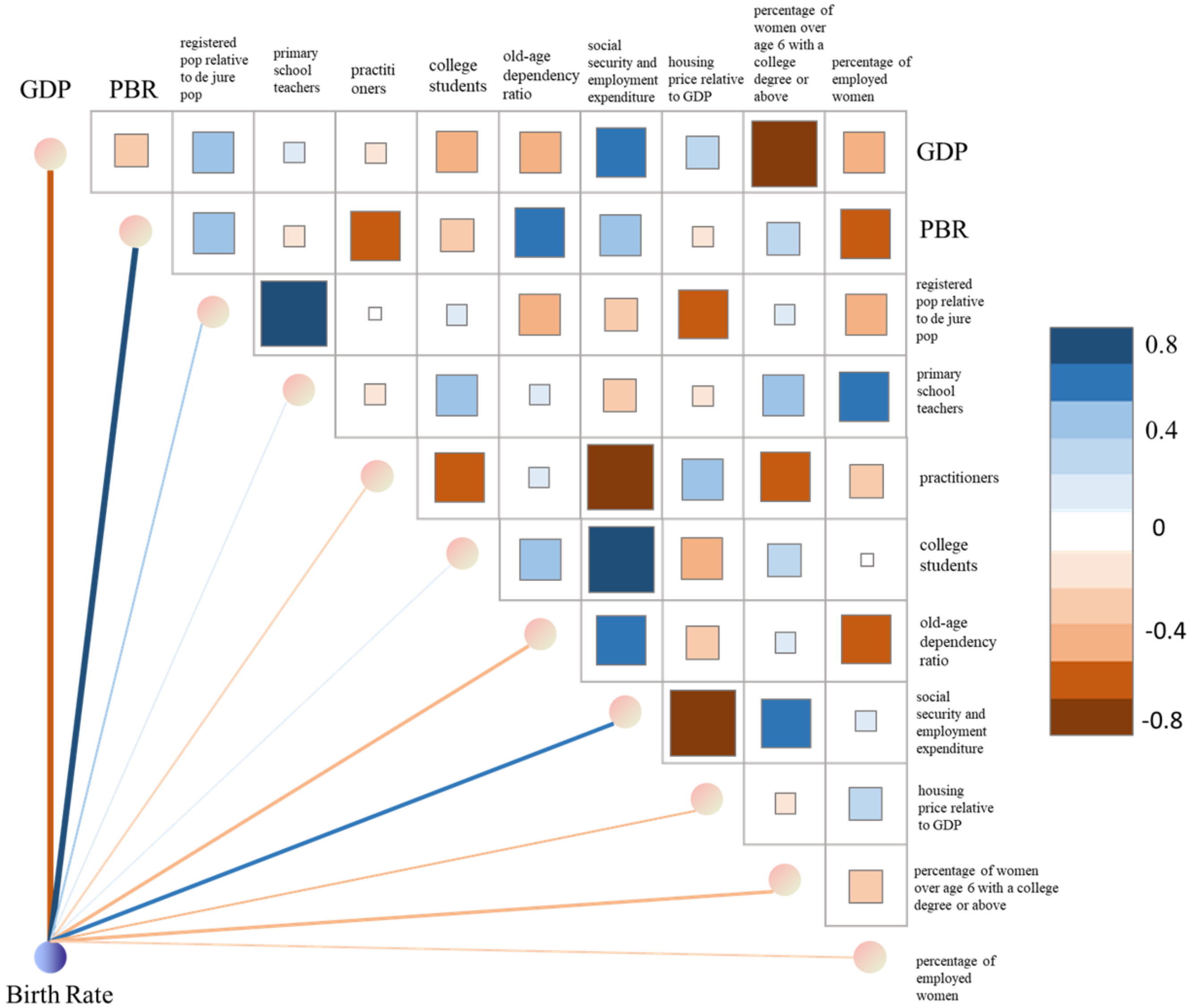


Fig 6. Heatmap of Correlation among Multi-dimensional Input Variables and Importance Assessment Plot.
